# Estimating healthcare expenditures after becoming divorced or widowed using propensity score matching

**DOI:** 10.1007/s10198-022-01532-z

**Published:** 2022-10-17

**Authors:** Iris Meulman, Bette Loef, Niek Stadhouders, Tron Anders Moger, Albert Wong, Johan J. Polder, Ellen Uiters

**Affiliations:** 1 Center for Health and Society, National Institute for Public Health and the Environment, P.O. Box 1, 3720 BA Bilthoven, The Netherlands; 2 Department Tranzo, Tilburg School of Social and Behavioral Sciences, Tilburg University, Tilburg, The Netherlands; 3 Center for Nutrition, Prevention and Health Services, National Institute for Public Health and the Environment, Bilthoven, The Netherlands; 4 Department of Health Management and Health Economics, University of Oslo, Oslo, Norway; 5 Department of Statistics, Informatics and Modeling, National Institute for Public Health and the Environment, Bilthoven, The Netherlands; 6 Scientific Center for Quality of Healthcare, Radboud University Medical Center, Nijmegen, the Netherlands

**Keywords:** Divorce, Healthcare expenditure, Healthcare utilization, Marital status change, Propensity score matching, Widowhood

## Abstract

Becoming divorced or widowed are stressful life events experienced by a substantial part of the population. While marital status is a significant predictor in many studies on healthcare expenditures, effects of a change in marital status, specifically becoming divorced or widowed, are less investigated. This study combines individual health claims data and registered sociodemographic characteristics from all Dutch inhabitants (about 17 million) to estimate the differences in healthcare expenditure for individuals whose marital status changed (*n* = 469,901) compared to individuals who remained married, using propensity score matching and generalized linear models. We found that individuals who were (long-term) divorced or widowed had 12–27% higher healthcare expenditures (RR = 1.12, 95% CI 1.11–1.14; RR = 1.27, 95% CI 1.26–1.29) than individuals who remained married. Foremost, this could be attributed to higher spending on mental healthcare and home care. Higher healthcare expenditures are observed for both divorced and widowed individuals, both recently and long-term divorced/widowed individuals, and across all age groups, income levels and educational levels.

**Supplementary Information:**

The online version contains supplementary material available at 10.1007/s10198-022-01532-z.

## Background

Marital status is a significant predictor in many studies on healthcare expenditure [1–3]. According to the Social Readjustment Scale of Holmes and Rahe [4], divorce and the death of a spouse are ranked as the most stressful life events. Marital dissolution may significantly disturb daily routines and accustomed life patterns and, therefore, require the most adjustment. Since the introduction of this scale in 1967, numerous studies have been devoted to these life events and their influence on well-being and health outcomes [5, 6]. For example, exposure to negative life events generally has a negative impact on happiness and subjective well-being [7–9] and increases the probability of developing depression, cardiovascular and infectious diseases [6, 10] and mortality [11].

Nevertheless, remarkably little is known about healthcare utilization or expenditure in the years following marital dissolution, particularly for divorce. The study by Prigerson, Maciejewski and Rosenheck [12] showed that recently divorced women consult a psychiatrist more often, but do not spend more nights in the hospital or visit a doctor more often compared to married women. Findings on healthcare utilization after widowhood are mixed. On the one hand, studies have indicated that spousal loss increases healthcare utilization or healthcare expenditures, varying by 18–59% higher [13–18]. The higher observed healthcare expenditures among widow(er)s were specifically assigned to increased long-term care, home care, hospital care and/or nursing homes expenditures [13, 16]. Furthermore, widowed individuals were more likely to have more hospital days when initially living in a two-person household and, therefore, were most likely to live alone after their spouse’s death [17]. These observations may suggest that the loss of informal care may potentially contribute to higher healthcare expenditures after marital dissolution. As informal care is especially relevant among the elderly, the differences in healthcare expenditures between individuals who have experienced a change in marital status and individuals who remained married are also hypothesized to increase with age. Besides, differences in healthcare expenditures would be lower among remarried individuals than individuals who did not remarry since the new spouse can provide the lacking informal care or necessary social support [19]. Contrarily, van Boekel, Cloin and Luijkx [20] and Wingen and Otten [21] showed that recent widowhood was negatively associated with psychological well-being and health, but found no impact on healthcare expenditure. This may probably be the result of the exclusion of long-term care expenditures and mental healthcare expenditures. When comparing the impact of divorce to widowhood, most studies found that, while controlling for age, becoming divorced has a smaller impact on several health outcomes than becoming widowed, e.g., a lower elevation in depressive symptoms [22], a smaller decline in subjective well-being [8] and, unlike widowhood, non-significant higher out-of-pocket medical spending [16].

Furthermore, the role of socioeconomic status in health outcomes and utilization after marital dissolution is yet unclear and inconclusive. Higher educated widow(er)s had a higher risk for inpatient psychiatric care, but not for outpatient psychiatric care and prescribed psychotropic medication [23]. Tseng, Petrie and Leon-Gonzalez [24] found that depressive symptoms may be worse among higher-income groups suggesting that income loss is higher among higher-income groups. However, McLeod and Kessler [25] concluded the opposite for income, education and occupation and psychological distress due to greater emotional vulnerability. Kung [26] found that wealth may positively affect widowers’ mental health, but not for widows, although the mechanism behind this finding remains unclear.

Differences in health outcomes and care use after marital dissolution between men and women are also inconclusive. In terms of healthcare utilization and expenditure after spousal death, Rolden et al. [13] found that the increase in expenditure was higher for men, while Ornstein et al. [14] found increased expenditure for women only and Oksuzyan, Jacobsen, Glaser et al. [27] found no difference between men and women in medication use and number of GP visits. Focusing on broader health outcomes, plenty of studies found that spousal loss affects both men and women, but the impact may be stronger among men [28, 29]. For example, in terms of life satisfaction [30], well-being [31], psychological distress[32], sensitivity to depressogenic effects [33] self-assessed health among elderly [34], morbidity and mortality [11, 29, 35]. However, more recent studies found no or inconsistent sex differences in the effect of marital dissolution on mental health [36–38] and mortality [39]. In addition, limited number of studies even find a stronger decline in health after marital dissolution among women: women had a higher probability of reporting declined self-assessed health after marital dissolution [40] and severe mental health status [41].

Given the high prevalence of divorce and widowhood and rising healthcare expenditures, the implication of marital dissolution may be of interest to policymakers as a source of potential gains in well-being and preventable healthcare expenditures, which may contribute to the sustainability of the healthcare system. Therefore, this study explores whether there are any differences in healthcare expenditures between individuals whose marriages have been dissolved and matched individuals who remained married (main analysis). To gain more insight into the relationship between becoming divorced or widowed and healthcare expenditure, several sub-analyses will be performed. This study additionally examines (a) differences between recent change in marital status and long-term divorce or widowhood, (b) differences between divorce or widowhood, (c) the variation by age, (d) the effect of remarriage, (e) the variation by socioeconomic status, (f) the variation by sex and (g) the attribution of specific types of care.

## Data and methods

### Data

This study concerns an observational study based on healthcare claims data from 2012 to 2017 and characteristics at the individual level covering the full population of the Netherlands (about 17 million inhabitants), in which individuals who were divorced or widowed were compared to individuals who remained married throughout using a propensity score matching (PSM) design and regression modeling. Healthcare expenditures, based on claims reimbursed by the health insurance companies, includes all types of care covered by the mandatory benefits package and any associated out-of-pocket payments due to cost-sharing. Because health insurance is mandatory in the Netherlands, this registry covers practically the entire Dutch population. Sociodemographic characteristics were derived from the personal records database (age, sex and marital status record) and the system of social–statistical files (highest level of completed education and standardized disposable household income). Data on the highest level of completed education for 2013 were available for only 50% of the population. Because the educational registry database was expanded retroactively, the highest level of completed education for 2017 was used if the information for 2013 was unavailable.

Statistics Netherlands functioned as a trusted third party, enabling the linkage between datasets, while ensuring the privacy of the involved individuals, according to Dutch law (Statistics Netherlands Act 2003). Sum of total healthcare expenditures from 2014 to 2017 were linked to marital status records from 2009 to 2017, individual sociodemographic characteristics from 2013 (age, sex, highest level of completed education, standardized disposable household income) and previous healthcare expenditures from 2012.

### Study population

Individuals were selected if the information on age, sex, marital status record, the highest level of completed education, standardized disposable household income and healthcare expenditures was available. For the research group, i.e., group consisting of individuals who became divorced or widowed, individuals were selected if they were (a) married for at least 4 years before getting divorced or widowed in 2013 and did not experience more than one change in marital status from 2014 to 2017 (‘recently divorced’ and ‘recently widowed’) (i.e., including individuals who remarried once) or (b) divorced or widowed in or before 2009 and their marital status remained unchanged from 2009 to 2017 (‘long-term divorced’ and ‘long-term widowed’). Individuals who were married in 2009 and their marital status remained unchanged from 2009 to 2017 functioned as the control group (‘long-term married’).

The study population was further restricted to individuals aged 25–79 years because of reasonable age for individuals to experience a change in marital status and, as a result, sufficient sample sizes. Moreover, the sample size was further complicated by the coverage of educational attainment among individuals over 80.

### Measures

#### Healthcare expenditure

The main outcome measure was the sum of total healthcare expenditures in euros for 2014 to 2017 covered by mandatory health insurance. This covers medical care provided by general practitioners (GP), medical specialists, obstetricians, remedial therapists, speech therapists or occupational therapists; home care (only short-term district nursing with medical necessity); hospitalization; mental healthcare for the first three years; physiotherapy for people with chronic illnesses; nutritional care; several types of medication; medical aids; and ambulance support and transport [42]. A deductible or co-payment could be required for some types of care, which was included in the healthcare expenditure data. Because homecare was covered by the mandatory benefit package from 2015, the sum of homecare expenditures comprised expenditures for 2015–2017. Maternity care only applied to women up to age 44 as maternity care is only used by women of childbearing age.

#### Marital status change

The two main determinants, as indicated before, were becoming divorced or widowed in 2013 and being divorced or widowed throughout 2009–2017. Individuals who met these criteria were identified based on annually registered marital status from 2009 to 2017. Marital status was operationalized as never married (excluded), married, divorced or widowed. In this typology, marriage included both statutorily married individuals and individuals with registered partnerships.

#### Matching variables

Age (five-year categories), sex (male/female), the highest level of completed education (low: primary education or lower; low–moderate: lower vocational education or lower secondary education; moderate–high: intermediate vocational education or higher secondary education; high: higher vocational education or university), standardized disposable household income (in quintiles) for 2013, and healthcare expenditures (in euros) for 2012 were included as matching variables. Healthcare expenditures in 2012 were used to control for healthcare expenditure prior to the follow-up period (i.e., baseline healthcare expenditure).

### Propensity score matching

To reduce any pre-existing differences in individual characteristics, divorced or widowed individuals were matched with individuals who remained married using one-to-one nearest neighbor propensity score matching (PSM) without replacement [43, 44]. The matching was based on age, sex, standardized disposable household income in 2013, the highest level of completed education for 2013 and healthcare expenditures in 2012. Balancing performance was assessed on the absolute standardized difference of means, the ratio of variance of the matching variables between research and control group and propensity score distribution [44].

PSM was conducted using MatchIt and covariate balance was assessed using cobalt, integrated MatchIt plot and summary statistics [45, 46] in R V.3.6.2. (R CoreTeam 2020).

### Statistical analysis

After PSM, regression analyses were performed on the matched sample to estimate differences in healthcare expenditures between individuals who experience a change in marital status and individuals who remained married. Generalized linear models (GLMs) with gamma distribution and log-link proved to be most suitable regression model due to the positively skewed sum of total healthcare expenditure [47], because nearly all individuals incurred any healthcare expenditure from 2014 to 2017 and according to the Akaike Information Criterion (AIC) and Bayesian Information Criterion (BIC). As a result, differences in healthcare expenditures are expressed in rate ratios (RR). To answer sub-questions a–c, a distinction between divorce or widowhood, between recent change in marital status or long-term divorced or widowed, and between age categories (25–44 years, 45–64 years and 65–79 years) was made. Additionally, analyses were stratified by remarriage within four years for the recently divorced or widowed individuals (sub-question d), by income quintile and educational attainment (sub-question e) and sex (sub-question f). For sub-question g, total healthcare expenditure was decomposed into, and analyzed separately for, expenditures for GP-care, medical specialist care, mental healthcare, pharmaceuticals, homecare and maternity care. Because a large proportion of individuals have no spending for these specific types of care, the probability of healthcare expenditure and the expenditure among users are analyzed in a two-stage model [48]. In the first part, the probability of healthcare expenditures for these types of care was analyzed using logistic regression and expressed in odds ratios (OR). In the second part, the conditional healthcare expenditures among users were analyzed using GLM with gamma distribution and log-link and expressed in rate ratios.

Statistical analyses were conducted using IBM SPSS Statistics, V.25.0 (IBM Corp, New York).

## Results

### Study population

**Fig. 1:**
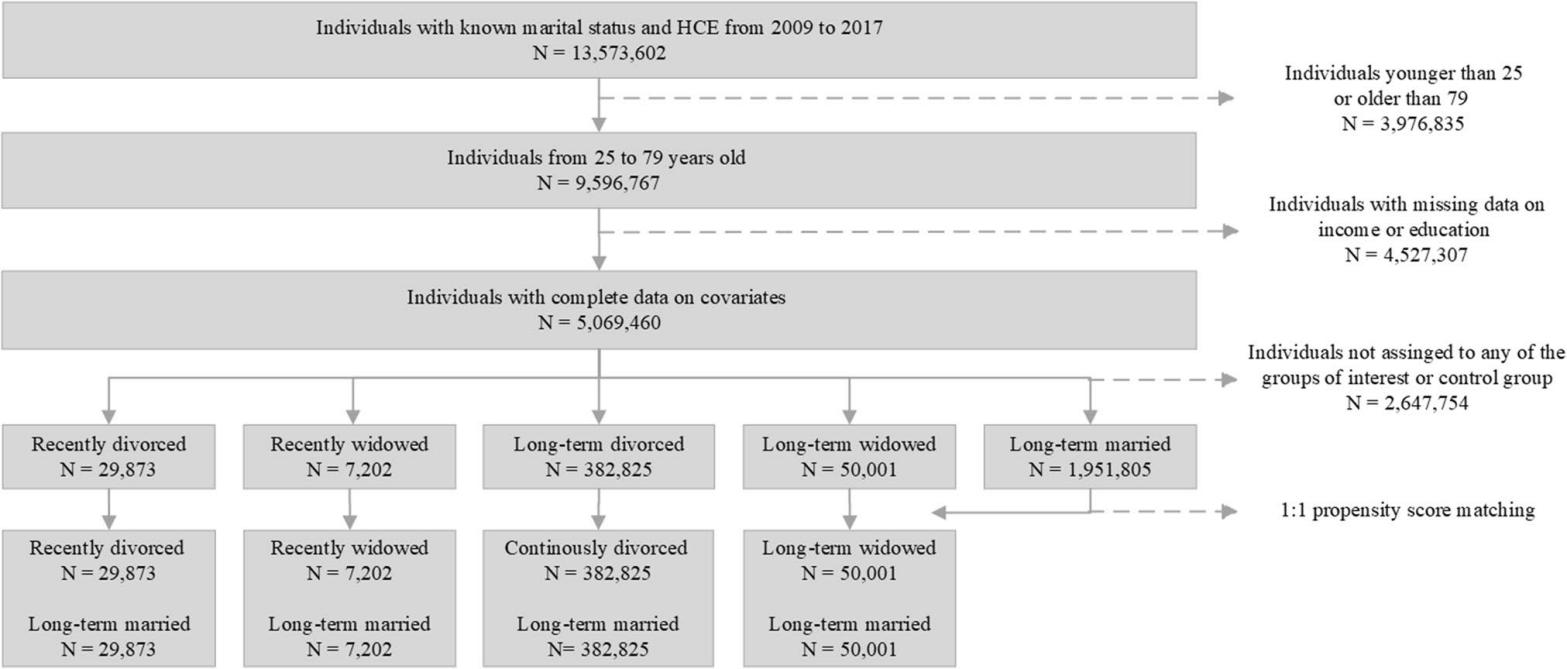
Flowchart of study population

Data on healthcare expenditures, sex, standardized disposable household income and the highest level of completed education were available for 5,069,460 individuals aged 25–79 years. Nearly 4 million individuals were excluded because they were under 25 or over 79 and 4.5 million individuals because of lacking data on income or education (Fig. 1). Individuals were categorized into ‘recently divorced (*n* = 29,873), ‘recently widowed’ (*n* = 7202), ‘long-term divorced’ (*n* = 382,825), ‘long-term widowed’ (*n* = 50,001) and ‘long-term married’ (*n* = 1,951,805) (Table 1). After one-to-one nearest neighbor PSM, the control group consisted of 469,901 long-term married individuals. Women were more likely to experience a change in marital status, especially widowhood (widowed: 69.0% female, long-term widowed: 76.8% female). Divorced individuals were more likely to remarry within four years after marital dissolution than widowed individuals (divorced: 12.5%, widowed: 4.5%).

**Table 1:** Characteristics of the study population

	Recently divorced^a^		Recently widowed^a^		Long-term divorced^b^		Long-term widowed^b^		Long-term married^b,c^	
	*N* or mean (SD)	% or median	*N* or mean (SD)	% or median	*N* or mean (SD)	% or median	*N* or mean (SD)	% or median	*N* or mean (SD)	% or median
Total	29,873	100	7202	100	382,825	100	50,001	100	469,901	100
Sex										
Male	13,307	44.5	2236	31.0	139,446	36.4	11,595	23.2	182,697	38.9
Female	16,566	55.5	4966	69.0	243,379	63.6	38,406	76.8	287,204	61.1
Age										
25–44	15,635	52.3	490	6.8	71,182	18.6	2007	4.0	89,314	19.0
45–64	13,863	46.4	4297	59.7	267,746	69.9	27,563	55.1	313,469	66.7
65–79	375	1.3	2415	33.5	43,897	11.5	20,431	40.9	67,118	14.3
Mean (SD) and median	44.3 (8.5)	44	60.1 (9.2)	61	53.1 (9.1)	53	61.9 (9.2)	62	53.5 (9.9)	53
Highest level of completed education										
Low	3428	11.5	1823	25.3	74,169	19.4	14,051	28.1	96,286	20.5
Low/moderate	4884	16.3	1668	23.2	80,758	21.1	12,188	24.4	96,652	20.6
Moderate/high	12,529	41.9	2414	33.5	146,527	38.3	16,080	32.2	177,061	37.7
High	9032	30.2	1297	18.0	81,371	21.3	7682	15.4	99,902	21.3
Standardized disposable household income										
1st quintile	5872	19.7	1258	17.5	126,128	32.9	9536	19.1	139,219	29.6
2nd quintile	5451	18.2	1601	22.2	84,886	22.2	11,337	22.7	108,025	23.0
3rd quintile	6301	21.1	1563	21.7	65,027	17.0	10,656	21.3	80,596	17.2
4th quintile	6418	21.5	1403	19.5	57,143	14.9	9866	19.7	74,234	15.8
5th quintile	5831	19.5	1377	19.1	49,641	13.0	8606	17.2	67,827	14.4
Healthcare expenditures in 2012										
Mean (SD) and median	€ 2206 (7150)	€ 500	€ 2362 (6552)	€ 735	€2543 (8375)	€ 678	€ 2641 (6697)	€ 877	€ 2446 (8649)	€ 627
Remarried within four years										
Yes	3727	12.5	326	4.5						
No	26,146	87.5	6876	95.5						

^a^Married for at least 4 years before getting divorced or widowed in 2013 and did not experience more than one change in marital status from 2014 to 2017

^b^Divorced, widowed or married in or before 2009 and their marital status remained unchanged from 2009 to 2017

^c^Control group after matching

The group that divorced in 2013 had the lowest healthcare expenditures in 2012, whereas the long-term widowed group had the highest healthcare expenditures in 2012. All groups of individuals who experienced a change in marital status had higher mean and median sum of total healthcare expenditures over 2014–2017 than the matched long-term married groups (Table 2).

**Table 2:** Sum of total healthcare expenditure 2014–2017 (outcome measure) per research group and corresponding control group

	Research group			Long-term married (control group)			Percentage difference in mean (%)
	Mean	SD	Median	Mean	SD	Median	
Full sample	€ 12,628	26,808	€ 4652	€ 10,560	28,299	€ 3872	20
Recently divorced or widowed	€ 10,332	23,437	€ 3730	€ 8670	22,563	€ 3036	19
Recently divorced	€ 9459	22,614	€ 3316	€ 7948	22,602	€ 2674	19
Recently widowed	€ 13,957	26,272	€ 5808	€ 11,663	22,153	€ 4862	20
Long-term divorced or widowed	€ 12,824	27,068	€ 4738	€ 10,722	28,731	€ 3950	20
Long-term divorced	€ 12,600	26,848	€ 4585	€ 10,447	28,591	€ 3773	21
Long-term widowed	€ 14,544	28,639	€ 5980	€ 12,822	29,698	€ 5415	13

### Differences in healthcare expenditure

Table 3 reports the regression results for the main analysis and sub-analyses a–c. Individuals who have experienced a change in marital status had 12–27% higher healthcare expenditures than the group that remained married. Among the individuals who recently became divorced or widowed, differences in healthcare expenditure in the first four years after marital dissolution ranged from 15 to 26%. The long-term divorced or widowed group had 12–28% higher healthcare expenditures from 2014 to 2017 compared to the group that remained married. Only individuals aged 25–44 who recently became widowed had no different healthcare expenditure than individuals who remained married (RR = 1.17, 95% CI 0.99–1.38), most likely suffering from small numbers (*n* = 490). No consistent differences were observed between divorce and widowhood. The differences in healthcare expenditures were greatest in the youngest age group.

**Table 3:** Rate ratios of sum of total healthcare expenditures from 2014 to 2017 for the groups that experienced a change in marital status compared to the group that remained married per age group

	Age 25–44		Age 45–64		Age 65–79	
	RR	95% CI	RR	95% CI	RR	95% CI
Full sample	1.27	1.26–1.29	1.20	1.20–1.21	1.12	1.11–1.14
Recently divorced or widowed	1.26	1.23–1.30	1.15	1.12–1.18	1.19	1.12–1.26
Recently divorced	1.27	1.23–1.31	1.13	1.09–1.16	1.20	1.01–1.42
Recently widowed	1.17	0.99–1.38	1.21	1.14–1.28	1.18	1.11–1.27
Long-term divorced or widowed	1.28	1.26–1.29	1.21	1.20–1.22	1.12	1.10–1.13
Long-term divorced	1.28	1.26–1.29	1.21	1.21–1.22	1.12	1.10–1.14
Long-term widowed	1.24	1.14–1.34	1.14	1.12–1.17	1.12	1.10–1.15

*RR* rate ratio, *95% CI* 95% confidence interval

### Differences in healthcare expenditure and the role of remarriage

**Fig. 2:**
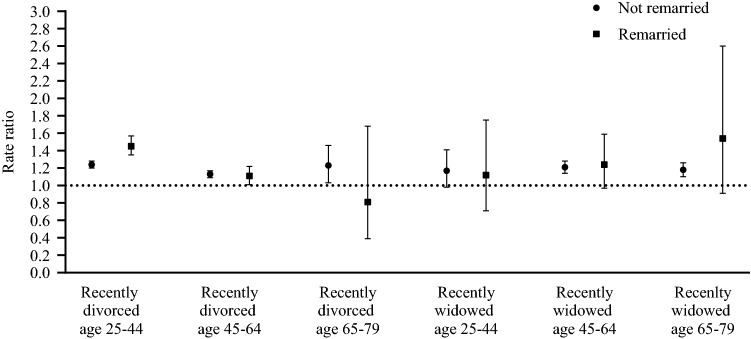
Differences in sum of total healthcare expenditures from 2014 to 2017 stratified by whether or not an individual remarried within 4 years after marital dissolution

To examine the role of remarriage on the difference in healthcare expenditures after becoming divorced or widowed, the results for recently divorced or widowed are stratified by whether or not individuals remarried within four years (sub-analysis d) (Fig. 2; Supplementary Appendix B Table 1). Healthcare expenditures among divorced or widowed individuals who did not remarry ranged from no difference to 24% higher (95% CI 1.20–1.28) than the group that remained married. Healthcare expenditures among divorced or widowed individuals who remarried ranged from no difference to 45% higher (95% CI 1.35–1.57). Compared to individuals who remained married, only individuals who remarried after the divorce between the age of 25–44 have different healthcare expenditures than the same group that has not remarried, with remarriage being associated with larger increases in health spending than remaining unmarried.

### Differences in healthcare expenditure and the variation by socioeconomic status

**Fig. 3:**
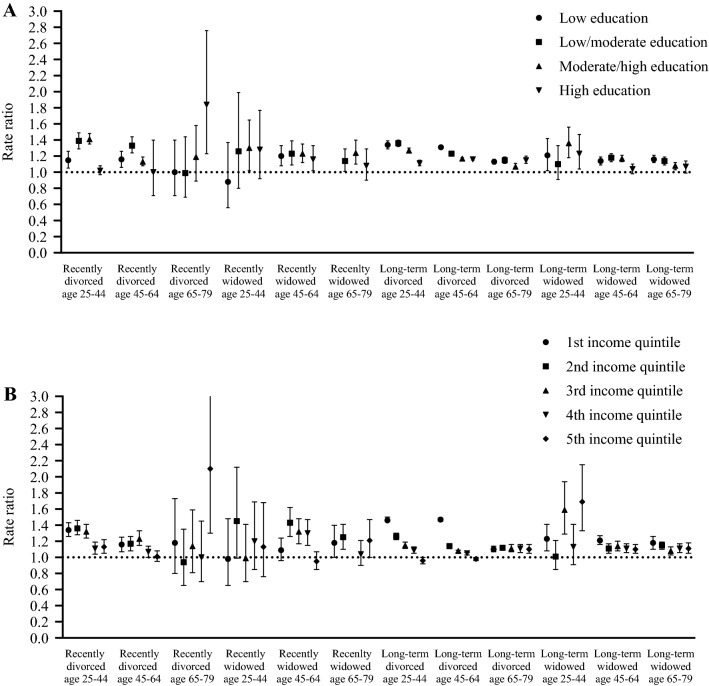
Differences in sum of total healthcare expenditures from 2014 to 2017 stratified by **A** highest level of completed education and **B** standardized disposable household income quintile

To examine the variation in differences in healthcare expenditure by socioeconomic status, results are stratified by educational attainment and standardized disposable household income (sub-analysis e) (Fig. 3; Supplementary Appendix B Table 1). Differences in healthcare expenditure between individuals who experienced a change in marital status and individuals who remained married ranged from no difference to 34% higher (95% CI 1.29–1.39) for individuals with low educational attainment and from no difference to 84% higher (95% CI 1.23–2.76) for individuals with high educational attainment. Differences in healthcare expenditures ranged from no difference to 47% higher (95% CI 1.45–1.49) for individuals in the lowest income quintile and from no difference to 110% higher (95% CI 1.30–3.38) for individuals in the highest income quintile. In the vast majority of groups who have experienced a change in marital status, healthcare expenditure was higher than the group that remained married across all income and educational levels. Although there is no consistent gradient in differences in healthcare expenditure between divorced or widowed individuals and individuals who remained married by income or education, highly educated and affluent had generally the smallest difference in healthcare expenditure. In addition, the dispersion varies widely between different age groups and recently/long-term divorced or widowed individuals.

### Differences in healthcare expenditure and the variation by sex

**Fig. 4:**
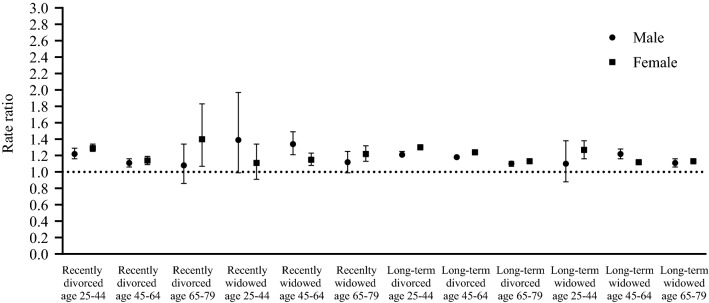
Differences in sum of total healthcare expenditure from 2014 to 2017 stratified by sex

Figure 4 shows the sex differences in healthcare expenditure after divorce and widowhood. Differences in healthcare expenditure between individuals who experienced a change in marital status and individuals who remained married ranged from no difference to 34% higher (95% CI 1.21–1.49) for men and from ranged from no difference to 40% higher (95% CI 1.07–1.83) for women (Supplementary Appendix B Table 1). Statistically significantly higher healthcare were observed for most groups who have experienced a change in marital status compared to individuals who remained married. Exceptions were recently divorced men of age 65–79, recently divorced men and women of age 25–44, recently divorced men of age 65–79 and long-term widowed men of age 25–44, who had no statistically significantly higher healthcare expenditure compared to their married counterparts.

### Differences in healthcare expenditure decomposed by type of healthcare

**Fig. 5:**
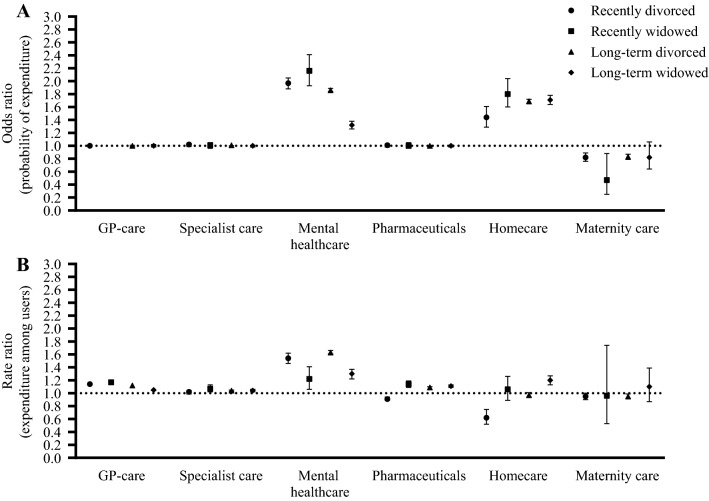
**A** Difference in the probability of expenditures for different types of healthcare (odds ratio) from 2014 to 2017 between the groups of individuals who experienced a change in marital status and the matched long-term married group **B** Difference in expenditures among users (rate ratio) for the groups of individuals who experienced a change in marital status and the group that remained married. Maternity care applies to women at the age of 25–44 only

Figure 5 depicts the variation in expenditure for different types of healthcare (GP-care, medical specialist care, mental healthcare, pharmaceuticals, homecare and maternity care) between the groups of individuals who experienced a change in marital status and the matched long-term married groups (sub-analysis g). Results are age-aggregated for clarity as no consistent variation by age was apparent for both the probability of and conditional healthcare expenditure (see Supplementary Appendix B Table 2 for all estimates by age group). Overall, divorced and widowed individuals had a higher probability of mental healthcare and homecare use, a lower probability of maternity care use (subsample of women aged 25–44) and no different probability of GP-care, medical specialist care and pharmaceutical utilization. Furthermore, for those who used care, mental healthcare expenditures were higher. Furthermore, expenditures for GP-care and pharmaceuticals was higher among both recently and long-term divorced and widowed users. Among users, recently divorced individuals have lower expenditures for pharmaceuticals and homecare than the matched long-term married individuals.

### Sensitivity analysis: quality of matching

In general, PSM strongly improved balance (see online Supplementary Appendix A Fig. 1, Fig. 2 and Table 1). As little imbalance may remain, additional adjustment for matching variables in the regression analysis, i.e., double adjustment, can control for any remaining imbalance [49]. Our double-adjustment analysis shows no major changes in effect estimators (see Supplementary Appendix B Table 3). Therefore, any remaining imbalance in matching variables had a limited impact on our results.

## Discussion

This study shows that individuals who have been divorced or widowed have consistently higher healthcare expenditures than individuals who remained married. Differences in four-year healthcare expenditures ranged from 12 to 27%. Further sub-analyses led to the following insights on healthcare expenditures after becoming divorced or widowed compared to remaining married: (a) no consistent differences were found between divorce and widowhood, (b) no consistent differences were found between a recent change in marital status and being long-term divorced or widowed, (c) among individuals aged 25–79 differences in healthcare expenditures between divorced or widowed individuals and individuals who remained married did not increase with age, (d) individuals who remarried after marital dissolution had similar differences in healthcare expenditure compared to individuals who remained married as individuals who did not remarry within four years, (e) healthcare expenditure of those who experienced a change in marital status are generally higher than those who remained married across all income and educational levels and (f) for both sexes, (g) differences in total healthcare expenditure between individuals who experienced a change in marital status and individuals who remained married could mainly be attributed to a higher probability of mental healthcare and home care use and a higher expenditures on mental healthcare conditional on use.

The estimates of 12–27% higher healthcare expenditures correspond to the previously described studies concluding on higher healthcare expenditures following widowhood [13–18]. Although several studies indicated that the health impact of becoming widowed was larger than becoming divorced [8, 16, 22], this study finds similar differences in healthcare expenditure among divorced and widowed individuals compared to individuals who remained married. This suggests that the loss of a spouse plays an important role in the higher healthcare expenditure, regardless of the cause of the loss.

The findings of higher healthcare expenditures among individuals who experienced a change in marital status hold for both recently and long-term divorced or widowed individuals shows the persistence of higher healthcare expenditure after marital dissolution. Long-lasting effects of a change in marital status have also been observed for the level of stress [50], life satisfaction [51], depressive symptoms and illness [52] after marital dissolution. Johnson and Wu [50] explained their findings of relatively constant and enduring elevated stress levels after a divorce using the social role theory. According to the social role theory, divorcees are facing more chronic everyday life stressors (e.g., social isolation, the stigma of divorce, economic hardship) and have lower levels of social support which cause them to be less able to cope with these stressors. The combination of continuous exposure to life stressors and lower capability of coping may cause healthcare expenditures to remain higher among divorcees many years after the life event itself. Likely, the social role theory may also contribute to the higher healthcare expenditure observed by this study. Furthermore, the findings of particularly higher chance of mental healthcare expenditures and home care, and higher expenditures for mental healthcare among users in this study also supports the mechanism that divorced and widowed individuals may suffer from poorer mental health and substitute spousal’s support and informal care with formal (home) care.

Similar differences in healthcare expenditure are found among individuals who have remarried within four years after marital dissolution and individuals who did not remarry compared to individuals who remained married. Nevertheless, divorced and widowed individuals who have remarried generally do not have statistically significant different healthcare expenditures than long-term married individuals. This corresponds to the findings of limited health differences between long-term married and individuals who remarry shortly after marital dissolution [19, 53, 54]. Potentially, remarriage may reduce part of the health risks and life stressors associated with marital dissolution, e.g., by providing social support and informal care or a better financial situation [55, 56]. However, individuals with lower healthcare expenditures may be more likely to remarry because healthier individuals may be more attractive and the potential gains of marriage are higher (i.e., a selection effect) [57], which cannot be ruled out with the current study design.

In general, the difference in healthcare expenditure between those who are divorced or widowed and those who remained married are in the same direction for all income and educational levels. For some age groups and marital status records, differences in healthcare expenditures seem to decline with higher educational attainment or income level, suggesting that low SES groups may be more vulnerable to these life events. On the other hand, other subgroups, especially older divorcees and younger widow(er)s, show inconsistent variation between socioeconomic groups and large degrees of dispersion. As these events are less frequent, the large dispersion can probably be explained by the smaller sample sizes of these groups. The overall inconsistent variation between socioeconomic levels found in this study is, however, not remarkable given the contradictory body of existing knowledge on the role of initial socioeconomic status on health outcomes and utilization after divorce or widowhood.

Furthermore, this study indicates that healthcare expenditure is generally higher for both men and women who have experienced a change in marital status. This corresponds to previous findings showing that men and women experience an overall decline in health outcomes after divorce and widowhood. The observed differences in healthcare expenditure in this study were more often insignificant for men and the rate ratio estimates were generally lower than those of women. Therefore, our findings suggest that the consequences of divorce and widowhood on healthcare expenditure are slightly more pronounced for women than men. This contradicts the bulk of literature stating that the health consequences of divorce and widowhood are generally more severe among men [28, 29] but agrees with a small number of more recent findings that also found opposite results [14, 40], indicating that the sex differences may have changed between generations. To confirm this hypothesis, further research is needed on current sex differences in health consequences and utilization following divorce and widowhood, and whether these differences have changed over time.

### Strengths and limitations

The use of registry data and propensity score matching limited the probability of influence of a selection effect. Because we have not investigated the underlying mechanisms, caution is necessary for concluding that higher healthcare expenditures are a direct result of divorce and widowhood. Due to the large sample size, it was possible to perform several sub-analyses among age, remarriage and socioeconomic groups.

An important limitation to consider is the possibility that pre-existing differences that were not controlled for, e.g., ethnicity [58], spousal age gap, number of children [59] or mental health status [60], may have confounded differences in healthcare expenditures as the number of matching variables was limited to five because of computational power. Because home care and short-term mental healthcare were transferred from the long-term care act to the health insurance act in 2015 [61], these forms of care were not included in the healthcare expenditure in 2012 used for PSM. This may bias the results because the reform may have affected the research and control groups differently ex ante since chronic users of home care or short-term mental healthcare are more likely to experience marital dissolution [60]. Furthermore, long-term care expenditure is not included as both matching variable and the outcome measure of total healthcare expenditure. Especially among the elderly, the use of long-term care is expected to increase after divorce or widowhood and may further magnify the observed differences. Most likely, the exclusion of long-term care expenditure explains why, contrary to our expectations, the differences in healthcare expenditure between divorced or widowed individuals and individuals who remained married did not increase with age. In addition, the use of long-term care can reduce the demand for GP-care and hospital admission [62, 63], implying a further underestimation of the differences between age groups. Another limitation is that the external validity may have been affected by the limited data available of the highest level of completed education up to and including 2013 (approximately 50%), mainly among the elderly and individuals who obtained their education from private institutions or internationally [64, 65]. Although it has been attempted to complement missing data with information collected in later years, remaining bias is possible if missing values concentrate for example among lower-educated elderly. Since the results for income point in the same direction, the missing data seems not selective or significantly influenced the results for education. Lastly, the classification into groups of interest and the control group required a minimum period of unchanged marital status. This requirement may have introduced two possible types of bias: selection bias and survivorship bias. The conditions of unchanged marital status before and after marital dissolution reduces the external validity and may possibly have influenced the findings. The interpretation of results only concerns a treatment effect for the subgroups that meet the study’s conditions, and not for the entire population of married individuals. Hypothetically, longer time periods may lead to more homogeneous groups and stronger findings, while shorter periods may lead to more heterogeneous groups and weaker findings. Survivorship bias, which is especially relevant among the elderly, is potentially created by the exclusion of individuals who died within four years (divorced or widowed in 2013) or seven years (long-term divorced or widowed) after marital dissolution. Excluding deceased could bias our results downwards, as mortality rates may increase upon widowhood [11, 17] and high (hospital) costs are associated with the last year of life [66]. Besides, the exclusion may have undermined a potential SES differences in mortality after widowhood [67] and, with that, associated healthcare expenditure.

### Policy implications and suggestions for further research

Despite the above-mentioned limitations, this study provides a comprehensive overview of healthcare expenditure after marital dissolution. It can function as a guideline for policy and further research. Preventative and supportive interventions targeted specifically at individuals who experience a change in marital status may contribute to reducing the differences in healthcare expenditure by marital status. As the results of this study suggest that poor mental health among divorced and widowed individuals may play a role in the observed higher healthcare expenditures, mental health should be an important attention point for policymakers. To further specify the policy target group, follow-up research may identify individual characteristics of divorced and widowed who are at increased risk of health loss and higher healthcare spending after marital dissolution. For example, changes in healthcare utilization after marital dissolution may depend on initial marital quality [12] or number of social contacts [68, 69].

## Conclusions

This study showed that becoming divorced or widowed is associated with increased healthcare spending, lasting for multiple years. This could mainly be attributed to a higher probability of mental care and home care use and higher expenditures on mental care conditional on use. Higher healthcare expenditures are observed after the loss of a partner regardless of whether the loss is caused by divorce or widowhood for both sexes and across all age groups, income levels and educational levels.

## Supplementary Information

Below is the link to the electronic supplementary material.

Supplementary file1 (DOCX 52 KB)

Supplementary file2 (DOCX 51 KB)

## Data Availability

Results are based on non-public microdata from Statistics Netherlands. Under certain conditions, these microdata are accessible for statistical and scientific research.
