# Supplementary material for: Estimating healthcare expenditures after becoming divorced or widowed using propensity score matching
Source: Eur J Health Econ. 2022 Oct 17;24(7):1047–60. doi: 10.1007/s10198-022-01532-z (PMC10406688; doi:10.1007/s10198-022-01532-z)
Supplement: Supplementary file 1 — Supplementary file1 (DOCX 52 KB) [file 10198_2022_1532_MOESM1_ESM.docx]

**Estimating healthcare expenditures after becoming divorced or widowed using propensity score matching**

**ONLINE APPENDIX A. Example of propensity score matching results**


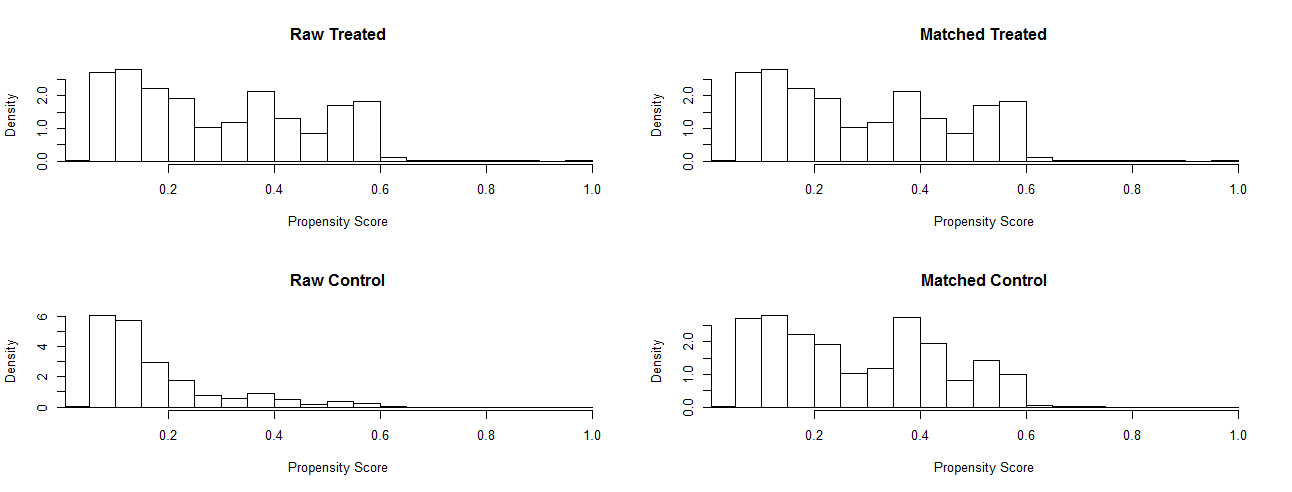


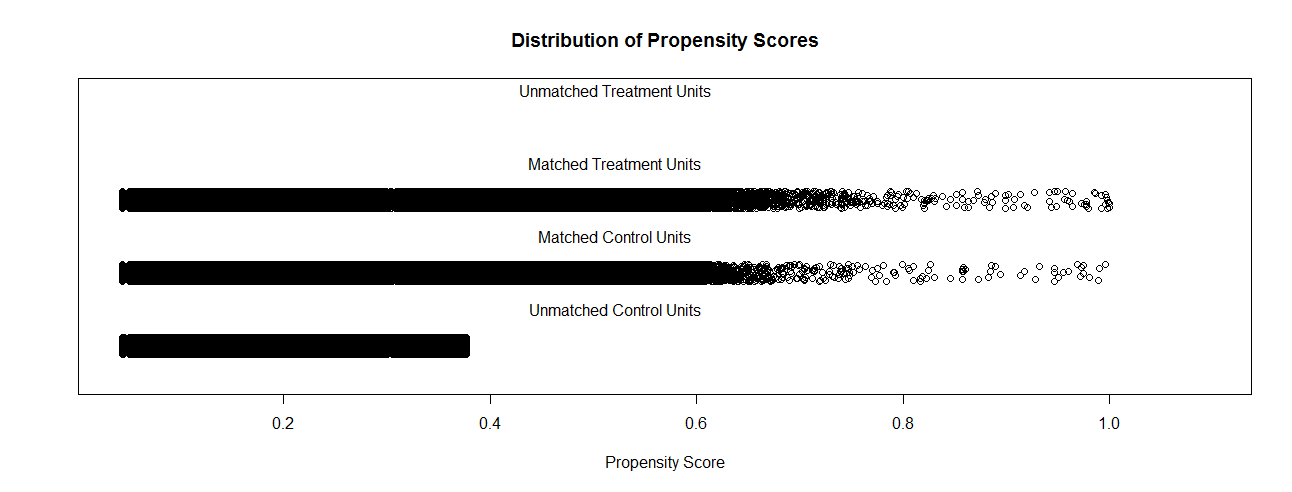


**Fig. 1** Distribution of the propensity scores before and after matching for long-term divorced individuals aged 45-64 (treatment group) and long-term married individuals aged 45-64 (control group) as example for matching result. (A) histogram of distrubtion, (B) jitterplot


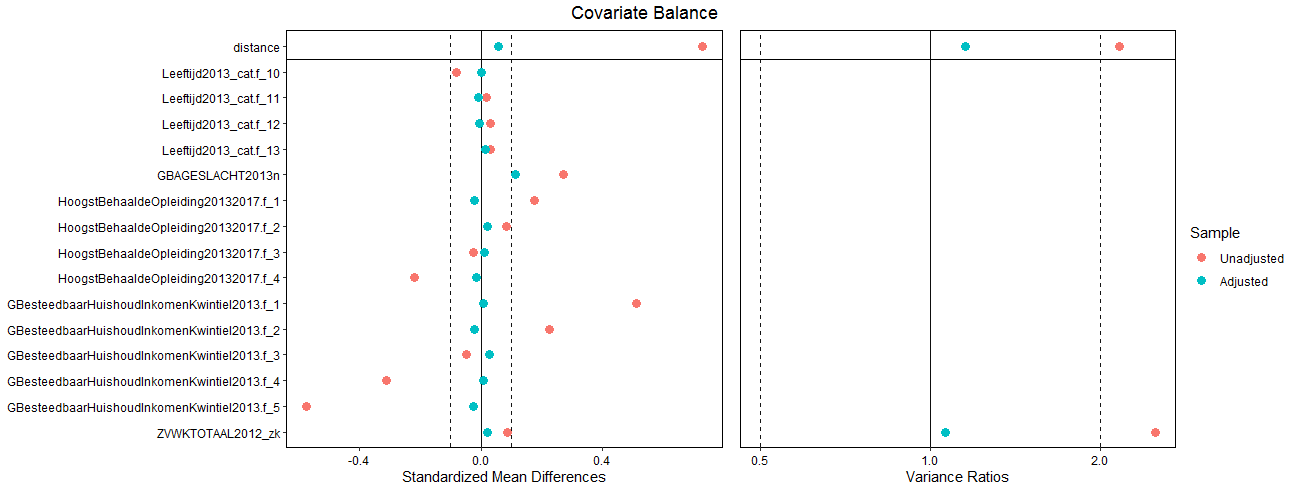


**Fig. 2** Covariate balance (standardized mean difference and variance ratios) before and after matching for long-term divorced individuals aged 45-64 compared to long-term married individuals aged 45-64 as an example of matching result

**Table 1**. Characteristics of study population before and after matching for long-term divorced individuals aged 45-64 and their control group (long-term married individuals aged 45-64) as example of matching result.

Notes: ^a^ divorced or married in or before 2009 and their marital status remained unchanged from 2009 to 2017, ^b^ control group, ^c^ percentage balance improvement before/after matching in mean difference between long-term divorced and long-term married

| **Age 45-64** | **Before matching** | | | | **After matching** | | | |  |
| --- | --- | --- | --- | --- | --- | --- | --- | --- | --- |
|  | Long-term divorced^a^ | | Long-term  Married^a,b^ | | Long-term divorced^a^ | | Long-term  married^a,b^ | | Balance imp.^c^ |
|  | N or  mean (SD) | % or median | N or  mean (SD) | % or median | N or  mean (SD) | % or median | N or  mean (SD) | % or median | % |
| **Total** | 267746 | 19.4 | 1110826 | 80.6 | 267746 | 50.00 | 267746 | 5000 | 92 |
| **Sex** |  |  |  |  |  |  |  |  |  |
| Male | 101691 | 38.0 | 568866 | 51.2 | 101691 | 38.0 | 116521 | 43.5 |  |
| Female | 166055 | 62.0 | 541960 | 48.8 | 166055 | 62.0 | 151225 | 56.5 | 58 |
| **Age** |  |  |  |  |  |  |  |  |  |
| Mean (SD) & median | 54.1 (5.5) | 54 | 53.6 (5.6) | 53 | 54.1 (5.5) | 54 | 54.0 (5.5) | 54 |  |
| 45-49 years | 67803 | 25.3 | 320149 | 28.8 | 67803 | 25.3 | 67684 | 25.3 | 99 |
| 50-54 years | 75580 | 28.2 | 304394 | 27.4 | 75580 | 28.2 | 76450 | 28.6 | 61 |
| 55-59 years | 69731 | 26.0 | 273663 | 24.6 | 69731 | 26.0 | 70472 | 26.3 | 80 |
| 60-64 years | 54632 | 20.4 | 212620 | 19.1 | 54632 | 20.4 | 53140 | 19.8 | 56 |
| **Highest completed education** | | | | | | | | | |
| Low | 50015 | 18.7 | 131134 | 11.8 | 50015 | 18.7 | 52156 | 19.5 |  |
| Low-moderate | 56040 | 20.9 | 194561 | 17.5 | 56040 | 20.9 | 53713 | 20.1 | 75 |
| Moderate-high | 103666 | 20.9 | 443617 | 17.5 | 103666 | 38.7 | 102338 | 38.2 | 59 |
| High | 267746 | 38.7 | 341514 | 39.9 | 58025 | 21.7 | 59539 | 22.2 | 94 |
| **Standardized disposable household income** | | | | | | | | | |
| 1^st^ quintile | 87478 | 32.7 | 96819 | 8.7 | 87478 | 32.7 | 86331 | 32.2 |  |
| 2^nd^ quintile | 54519 | 20.4 | 125978 | 11.3 | 54519 | 20.4 | 56707 | 21.2 | 91 |
| 3^rd^ quintile | 46451 | 17.3 | 212974 | 19.2 | 46451 | 17.3 | 43698 | 16.3 | 44 |
| 4^th^ quintile | 42056 | 15.7 | 299795 | 27.0 | 42056 | 15.7 | 41408 | 15.5 | 98 |
| 5^th^ quintile | 37242 | 13.9 | 375260 | 33.8 | 37242 | 13.9 | 39602 | 14.8 | 96 |
| **Healthcare expenditure 2012** | | | | | | | | | |
| Mean (SD) & median | 2534 (8840) | 660 | 1769 (5581) | 427 | 2534 (8840) | 660 | 2353 (8572) | 580 | 76 |
