# Supplementary material for: Estimating healthcare expenditures after becoming divorced or widowed using propensity score matching
Source: Eur J Health Econ. 2022 Oct 17;24(7):1047–60. doi: 10.1007/s10198-022-01532-z (PMC10406688; doi:10.1007/s10198-022-01532-z)
Supplement: Supplementary file 2 — Supplementary file2 (DOCX 51 KB) [file 10198_2022_1532_MOESM2_ESM.docx]

**Estimating healthcare expenditures after becoming divorced or widowed using propensity score matching**

**ONLINE APPENDIX B. Additional tables**

**Table 1.** Rate ratios of sum of total healthcare expenditures 2014-2017 for divorced/widowed compared to long-term married counterparts stratified by remarriage, educational attainment and household income per age group. N = number of individuals, RR = rate ratio, 95% CI = 95% confidence interval)

Note: ^a^ unable to fit GLM.

|  | Age 25-44 | | | | | | Age 45-64 | | | | Age 65-79 | | | |
| --- | --- | --- | --- | --- | --- | --- | --- | --- | --- | --- | --- | --- | --- | --- |
|  | N | | | % | RR | 95% CI | N | % | RR | 95% CI | N | % | RR | 95% CI |
| **Recently divorced** | | |  |  |  |  |  |  |  |  |  |  |  |  |
| Remarried |  | | |  |  |  |  |  |  |  |  |  |  |  |
| *No* | 13451 | | | 86.0 | 1.24 | 1.20-1.28 | 12338 | 89.0 | 1.13 | 1.09-1.17 | 357 | 95.2 | 1.23 | 1.03-1.46 |
| *Yes* | 2184 | | | 14.0 | 1.45 | 1.35-1.57 | 1525 | 11.0 | 1.11 | 1.01-1.22 | 18 | 4.8 | 0.81 | 0.39-1.68 |
| Education |  | | |  |  |  |  |  |  |  |  |  |  |  |
| *Low* | 1487 | | | 9.51 | 1.15 | 1.05-1.26 | 1846 | 13.3 | 1.16 | 1.06-1.26 | 95 | 25.3 | 1.00 | 0.71-1.40 |
| *Low/moderate* | 2435 | | | 15.6 | 1.39 | 1.29-1.49 | 2385 | 17.2 | 1.33 | 1.24-1.44 | 64 | 17.1 | 0.99 | 0.69-1.44 |
| *Moderate/high* | 6831 | | | 43.7 | 1.41 | 1.35-1.48 | 5555 | 40.1 | 1.13 | 1.08-1.19 | 143 | 38.1 | 1.19 | 0.89-1.58 |
| *High* | 4882 | | | 31.2 | 1.02 | 0.97-1.08 | 4077 | 29.4 | 0.95 | 0.89-1.01 | 73 | 19.5 | 1.84 | 1.23-2.76 |
| Income |  | | |  |  |  |  |  |  |  |  |  |  |  |
| *1st quintile* | 3366 | | | 21.5 | 1.34 | 1.26-1.43 | 2433 | 17.6 | 1.16 | 1.07-1.25 | 73 | 19.5 | 1.18 | 0.80-1.73 |
| *2nd quintile* | 3042 | | | 19.5 | 1.36 | 1.28-1.46 | 2320 | 16.7 | 1.17 | 1.08-1.26 | 89 | 23.7 | 0.94 | 0.65-1.35 |
| *3rd quintile* | 3405 | | | 21.8 | 1.32 | 1.24-1.41 | 2817 | 20.3 | 1.23 | 1.15-1.33 | 79 | 21.1 | 1.14 | 0.81-1.59 |
| *4th quintile* | 3193 | | | 20.4 | 1.11 | 1.04-1.19 | 3151 | 22.7 | 1.07 | 1.00-1.14 | 74 | 19.7 | 1.00 | 0.70-1.45 |
| *5th quintile* | 2629 | | | 16.8 | 1.13 | 1.05-1.22 | 3142 | 22.7 | 1.01 | 0.95-1.08 | 60 | 16.0 | 2.10 | 1.30-3.38 |
| Sex |  | | |  |  |  |  |  |  |  |  |  |  |  |
| *Male* | 6095 | | | 39.0 | 1.22 | 1.16-1.29 | 6997 | 50.5 | 1.11 | 1.06-1.16 | 215 | 57.3 | 1.08 | 0.86-1.34 |
| *Female* | 9540 | | | 61.0 | 1.29 | 1.25-1.34 | 6866 | 49.5 | 1.14 | 1.09-1.19 | 160 | 42.7 | 1.40 | 1.07-1.83 |
|  |  | | |  |  |  |  |  |  |  |  |  |  |  |
| **Recently widowed** | |  | |  |  |  |  |  |  |  |  |  |  |  |
| Remarried |  | | |  |  |  |  |  |  |  |  |  |  |  |
| *No* | 434 | | | 88.6 | 1.17 | 0.98-1.41 | 4069 | 94.7 | 1.21 | 1.14-1.28 | 2373 | 98.3 | 1.18 | 1.10-1.26 |
| *Yes* | 56 | | | 11.4 | 1.12 | 0.71-1.75 | 228 | 46.5 | 1.24 | 0.97-1.59 | 42 | 8.6 | 1.54 | 0.91-2.60 |
| Education |  | | |  |  |  |  |  |  |  |  |  |  |  |
| *Low* | 77 | | | 15.7 | 0.88 | 0.56-1.37 | 817 | 19.0 | 1.20 | 1.08-1.33 | 364 | 15.1 | ^a^ | ^a^ |
| *Low/moderate* | 108 | | | 22.0 | 1.26 | 0.80-1.99 | 789 | 18.4 | 1.23 | 1.09-1.39 | 704 | 29.2 | 1.14 | 1.01-1.29 |
| *Moderate/high* | 107 | | | 21.8 | 1.30 | 1.02-1.65 | 870 | 20.2 | 1.23 | 1.12-1.35 | 586 | 24.3 | 1.24 | 1.10-1.40 |
| High | 103 | | | 21.0 | 1.28 | 0.92-1.77 | 853 | 19.9 | 1.16 | 1.02-1.33 | 447 | 18.5 | 1.08 | 0.90-1.29 |
| Income |  | | |  |  |  |  |  |  |  |  |  |  |  |
| *1st quintile* | 77 | | | 15.7 | 0.98 | 0.65-1.48 | 817 | 19.0 | 1.09 | 0.96-1.24 | 364 | 15.1 | 1.18 | 1.00-1.40 |
| *2nd quintile* | 108 | | | 22.0 | 1.45 | 0.99-2.12 | 789 | 18.4 | 1.43 | 1.26-1.62 | 704 | 29.2 | 1.25 | 1.10-1.41 |
| *3rd quintile* | 107 | | | 21.8 | 0.99 | 0.70-1.41 | 870 | 20.2 | 1.32 | 1.17-1.48 | 586 | 24.3 | ^a^ | ^a^ |
| *4th quintile* | 103 | | | 21.0 | 1.20 | 0.85-1.69 | 853 | 19.9 | 1.30 | 1.15-1.47 | 447 | 18.5 | 1.04 | 0.90-1.21 |
| *5th quintile* | 95 | | | 19.4 | 1.13 | 0.76-1.68 | 968 | 22.5 | 0.95 | 0.85-1.07 | 314 | 13.0 | 1.21 | 1.00-1.47 |
| Sex |  | | |  |  |  |  |  |  |  |  |  |  |  |
| *Male* | 131 | | | 26.7 | 1.39 | 0.99-1.97 | 1313 | 30.6 | 1.34 | 1.21-1.49 | 792 | 32.8 | 1.12 | 0.99-1.25 |
| *Female* | 359 | | | 73.3 | 1.11 | 0.91-1.34 | 2984 | 69.4 | 1.15 | 1.08-1.23 | 1623 | 67.2 | 1.22 | 1.13-1.32 |
|  |  | | |  |  |  |  |  |  |  |  |  |  |  |
| **Long-term divorced** | | | |  |  |  |  |  |  |  |  |  |  |  |
| Education |  | | |  |  |  |  |  |  |  |  |  |  |  |
| *Low* | 10237 | | | 14.4 | 1.34 | 1.29-1.39 | 50015 | 18.7 | 1.31 | 1.29-1.33 | 13917 | 31.7 | 1.13 | 1.10-1.16 |
| *Low/moderate* | 14818 | | | 20.8 | 1.36 | 1.32-1.40 | 56040 | 20.9 | 1.23 | 1.21-1.25 | 9900 | 22.6 | 1.15 | 1.11-1.19 |
| *Moderate/high* | 30709 | | | 43.1 | 1.27 | 1.24-1.30 | 103666 | 38.7 | 1.17 | 1.15-1.18 | 12152 | 27.7 | 1.07 | 1.0-4-1.11 |
| *High* | 15418 | | | 21.7 | 1.11 | 1.08-1.15 | 58025 | 21.7 | 1.16 | 1.15-1.18 | 7928 | 18.1 | 1.15 | 1.11-1.20 |
| Income |  | | |  |  |  |  |  |  |  |  |  |  |  |
| *1st quintile* | 25607 | | | 36.0 | 1.46 | 1.43-1.50 | 87478 | 32.7 | 1.47 | 1.45-1.49 | 13043 | 29.7 | 1.10 | 1.07-1.14 |
| *2nd quintile* | 15827 | | | 22.2 | 1.26 | 1.22-1.30 | 54519 | 20.4 | 1.14 | 1.12-1.16 | 14540 | 33.1 | 1.12 | 1.09-1.15 |
| *3rd quintile* | 11842 | | | 16.6 | 1.15 | 1.11-1.19 | 46451 | 17.3 | 1.08 | 1.06-1.09 | 6734 | 15.3 | 1.11 | 1.07-1.16 |
| *4th quintile* | 9682 | | | 13.6 | 1.09 | 1.05-1.13 | 42056 | 15.7 | 1.05 | 1.03-1.06 | 5405 | 12.3 | 1.11 | 1.06-1.16 |
| *5th quintile* | 8224 | | | 11.6 | 0.96 | 0.92-1.00 | 37242 | 13.9 | 0.98 | 0.96-1.00 | 4175 | 9.5 | 1.10 | 1.05-1.16 |
| Sex |  | | |  |  |  |  |  |  |  |  |  |  |  |
| *Male* | 21752 | | | 30.6 | 1.21 | 1.18-1.25 | 101691 | 38.0 | 1.18 | 1.17-1.19 | 16003 | 36.5 | 1.10 | 1.07-1.13 |
| *Female* | 49430 | | | 69.4 | 1.30 | 1.28-1.32 | 166055 | 62.0 | 1.24 | 1.23-1.25 | 27894 | 63.5 | 1.13 | 1.11-1.16 |
|  |  | | |  |  |  |  |  |  |  |  |  |  |  |
| **Long-term widowed** | | | |  |  |  |  |  |  |  |  |  |  |  |
| Education |  | | |  |  |  |  |  |  |  |  |  |  |  |
| *Low* | 438 | | | 21.8 | 1.21 | 1.02-1.42 | 6782 | 24.6 | 1.14 | 1.09-1.19 | 6831 | 33.4 | 1.16 | 1.12-1.21 |
| *Low/moderate* | 368 | | | 18.3 | 1.10 | 0.91-1.33 | 6209 | 22.5 | 1.18 | 1.13-1.23 | 5611 | 27.5 | 1.14 | 1.09-1.19 |
| *Moderate/high* | 704 | | | 35.1 | 1.36 | 1.18-1.56 | 9694 | 35.2 | 1.17 | 1.13-1.21 | 5682 | 27.8 | 1.08 | 1.03-1.12 |
| *High* | 497 | | | 24.8 | 1.23 | 1.04-1.47 | 4878 | 17.7 | 1.04 | 0.98-1.10 | 2307 | 11.3 | 1.07 | 0.99-1.14 |
| Income |  | | |  |  |  |  |  |  |  |  |  |  |  |
| *1st quintile* | 703 | | | 35.0 | 1.23 | 1.08-1.41 | 6633 | 24.1 | 1.21 | 1.16-1.27 | 2200 | 10.8 | 1.18 | 1.10-1.26 |
| *2nd quintile* | 446 | | | 22.2 | 1.01 | 0.85-1.21 | 4897 | 17.8 | 1.11 | 1.05-1.17 | 5994 | 29.3 | 1.15 | 1.10--1.19 |
| *3rd quintile* | 336 | | | 16.7 | 1.59 | 1.29-1.94 | 5341 | 19.4 | 1.14 | 1.08-1.20 | 4979 | 24.4 | 1.08 | 1.03-1.13 |
| *4th quintile* | 270 | | | 13.5 | 1.13 | 0.91-1.41 | 5447 | 19.8 | 1.11 | 1.06-1.17 | 4149 | 20.3 | 1.11 | 1.06-1.17 |
| *5th quintile* | 252 | | | 12.6 | 1.69 | 1.33-2.15 | 5245 | 19.0 | 1.10 | 1.05-1.16 | 3109 | 15.2 | 1.11 | 1.05-1.18 |
| Sex |  | | |  |  |  |  |  |  |  |  |  |  |  |
| *Male* | 373 | | | 18.6 | 1.10 | 0.88-1.38 | 6470 | 23.5 | 1.22 | 1.16-1.28 | 4752 | 23.3 | 1.11 | 1.06-1.16 |
| *Female* | 1634 | | | 81.4 | 1.27 | 1.16-1.38 | 21093 | 76.5 | 1.12 | 1.09-1.14 | 15679 | 76.7 | 1.13 | 1.10-1.16 |

**Table 2.** Decomposition of sum of total healthcare expenditures by GP-care, specialist care and mental healthcare for divorced/widowed compared to the matched long-term married group by age group. OR = odds ratio, RR = rate ratio, 95% CI = 95% confidence interval. Notes: ^a^ maternity care is only applicable for women age 25-44, ^b^ limited sample size or interindividual variation, and therefore insufficient statistical power

|  | All ages | | | | Age 25-44 | | | | Age 45-64 | | | | Age 65-79 | | | |
| --- | --- | --- | --- | --- | --- | --- | --- | --- | --- | --- | --- | --- | --- | --- | --- | --- |
|  | OR | 95% CI | RR | 95% CI | OR | 95% CI | RR | 95% CI | OR | 95% CI | RR | 95% CI | OR | 95% CI | RR | 95% CI |
| **Divorced** | | | | | | | | | | | | | | | | |
| GP-care | 1.00 | 0.98-1.02 | 1.14 | 1.13-1.16 | 1.00 | 0.97-1.03 | 1.18 | 1.16-1.19 | ^b^ | ^b^ | 1.11 | 1.09-1.13 | ^b^ | ^b^ | 1.09 | 0.99-1.21 |
| Specialist care | 1.02 | 0.99-1.04 | 1.02 | 0.99-1.04 | 1.02 | 0.99-1.06 | 1.07 | 1.04-1.11 | 1.01 | 0.97-1.04 | 0.98 | 0.95-1.02 | 1.00 | 0.81-1.23 | 0.92 | 0.76-1.12 |
| Mental healthcare | 1.97 | 1.88-2.05 | 1.54 | 1.46-1.62 | 1.98 | 1.87-2.09 | 1.45 | 1.36-1.56 | 1.94 | 1.82-2.08 | 1.61 | 1.48-1.75 | 2.90 | 1.61-5.21 | 8.63 | 3.60-20.65 |
| Pharmaceuticals | 1.01 | 0.98-1.03 | 0.91 | 0.89-0.94 | 1.01 | 0.98-1.04 | 0.90 | 0.87-0.93 | 1.00 | 0.97-1.04 | 0.92 | 0.89-0.95 | ** | **-** | 1.05 | 0.88-1.25 |
| Homecare | 1.44 | 1.29-1.61 | 0.62 | 0.52-0.75 | 1.40 | 1.14-1.72 | 0.77 | 0.55-1.07 | 1.44 | 1.25-1.66 | 0.01 | 0.00-0.81 | 1.63 | 1.04-2.56 | 1.27 | 0.69-2.31 |
| Maternity care | ^a^ | ^a^ | ^a^ | ^a^ | 0.82 | 0.76-0.89 | 0.95 | 0.90-1.00 | ^a^ | ^a^ | ^a^ | ^a^ | ^a^ | ^a^ | ^a^ | ^a^ |
| **Widowed** | | | | | | | | | | | | | | | | |
| GP-care | ^b^ | ^b^ | 1.17 | 1.14-1.19 | ^b^ | ^b^ | 1.26 | 1.16-1.38 | ^b^ | ^b^ | 1.19 | 1.15-1.22 | ^b^ | ^b^ | 1.12 | 1.08-1.17 |
| Specialist care | 1.01 | 0.96-1.05 | 1.07 | 1.02-1.13 | 1.01 | 0.84-1.21 | 1.02 | 0.84-1.23 | 1.01 | 0.95-1.07 | 1.08 | 1.01-1.14 | 1.00 | 0.92-1.08 | 1.08 | 1.00-1.16 |
| Mental healthcare | 2.16 | 1.93-2.41 | 1.22 | 1.06-1.41 | 2.13 | 1.54-2.93 | 1.44 | 1.00-2.09 | 2.34 | 2.03-2.69 | 1.06 | 0.89-1.26 | 1.66 | 1.30-2.12 | 1.84 | 1.32-2.55 |
| Pharmaceuticals | 1.00 | 0.96-1.05 | 1.14 | 1.09-1.19 | 1.00 | 0.84-1.20 | 0.91 | 0.75-1.10 | 1.00 | 0.94-1.07 | 1.20 | 1.13-1.28 | 1.01 | 0.93-1.09 | 1.07 | 1.00-1.15 |
| Homecare | 1.80 | 1.60-2.04 | 1.06 | 0.89-1.26 | ^b^ | ^b^ | ^b^ | ^b^ | 2.04 | 1.67-2.50 | 1.00 | 0.74-1.36 | 1.70 | 1.45-1.99 | 1.09 | 0.88-1.36 |
| Maternity care | ^a^ | ^a^ | ^a^ | ^a^ | 0.46 | 0.24-0.89 | 0.96 | 0.53-1.74 | ^a^ | ^a^ | ^a^ | ^a^ | ^a^ | ^a^ | ^a^ | ^a^ |
| **Long-term divorced** | | | | | | | | | | | | | | | | |
| GP-care | 1.00 | 0.99-1.01 | 1.12 | 1.12-1.12 | 1.00 | 0.98-1.01 | 1.15 | 1.14-1.16 | 1.00 | 0.99-1.01 | 1.12 | 1.11-1.12 | 1.00 | 0.98-1.02 | 1.09 | 1.08-1.10 |
| Specialist care | 1.01 | 1.00-1.01 | 1.04 | 1.03-1.05 | 1.02 | 1.01-1.04 | 1.04 | 1.03-1.06 | 1.00 | 1.00-1.01 | 1.05 | 1.04-1.06 | 0.99 | 0.97-1.01 | 1.01 | 0.99-1.03 |
| Mental healthcare | 1.86 | 1.84-1.89 | 1.63 | 1.61-1.66 | 1.88 | 1.83-1.93 | 1.57 | 1.52-1.62 | 1.83 | 1.80-1.86 | 1.66 | 1.63-1.70 | 2.25 | 2.13-2.39 | 1.51 | 1.40-1.64 |
| Pharmaceuticals | 1.00 | 0.99-1.01 | 1.09 | 1.09-1.10 | 1.01 | 0.99-1.02 | 1.00 | 0.98-1.02 | 1.00 | 0.99-1.01 | 1.13 | 1.12-1.14 | 0.99 | 0.97-1.01 | 1.02 | 1.01-1.04 |
| Homecare | 1.69 | 1.65-1.72 | 0.97 | 0.94-1.01 | 1.55 | 1.43-1.67 | 1.02 | 0.89-1.17 | 1.73 | 1.69-1.78 | 0.93 | 0.89-0.97 | 1.62 | 1.56-1.69 | 1.05 | 0.99-1.11 |
| Maternity care | ^a^ | ^a^ | ^a^ | ^a^ | 0.89 | 0.85-0.93 | 0.95 | 0.92-0.99 | ^a^ | ^a^ | ^a^ | ^a^ | ^a^ | ^a^ | ^a^ | ^a^ |
| **Long-term widowed** | | | | | | | | | | | | | | | | |
| GP-care | 1.00 | 0.98-1.02 | 1.05 | 1.04-1.05 | ^b^ | ^b^ | 1.06 | 1.01-1.10 | 1.00 | 0.98-1.02 | 1.04 | 1.03-1.05 | 1.00 | 0.97-1.03 | 1.05 | 1.04-1.06 |
| Specialist care | 1.00 | 0.98-1.01 | 1.04 | 1.02-1.06 | 1.01 | 0.92-1.10 | 1.06 | 0.97-1.16 | 1.00 | 0.97-1.02 | 1.05 | 1.03-1.08 | 0.99 | 0.97-1.02 | 1.03 | 1.00-1.05 |
| Mental healthcare | 1.32 | 1.26-1.38 | 1.30 | 1.22-1.37 | 1.51 | 1.28-1.77 | 1.26 | 1.05-1.52 | 1.36 | 1.28-1.43 | 1.36 | 1.27-1.46 | 1.17 | 1.07-1.28 | 1.20 | 1.05-1.36 |
| Pharmaceuticals | 1.00 | 0.98-1.01 | 1.11 | 1.09-1.13 | 0.99 | 0.91-1.09 | 1.34 | 1.21-1.48 | 1.00 | 0.97-1.02 | 1.16 | 1.13-1.18 | 1.00 | 0.97-1.02 | 1.06 | 1.03-1.08 |
| Homecare | 1.71 | 1.64-1.78 | 1.20 | 1.13-1.27 | 1.40 | 0.87-2.25 | 1.81 | 0.88-3.70 | 1.90 | 1.75-2.05 | 1.02 | 0.91-1.15 | 1.64 | 1.55-1.72 | 1.28 | 1.19-1.37 |

**Table 3.** Double-adjustment analysis. Crude model after PSM without further corrections. The double-adjusted model additionally corrects for matching variables (sex, age, interaction between sex and age, highest level of completed education, standardized disposable household income and healthcare expenditures in 2012). Expressed in rate ratios compared to long-term married. RR = rate ratio, 95% CI = 95% confidence interval.

Note: ^a^ unable to fit GLM.

|  | RR | 95% CI | Double adj. RR | 95% CI |
| --- | --- | --- | --- | --- |
| *Age 25-44* |  |  |  |  |
| **Entire sample** | 1.27 | 1.26-1.29 | 1.36 | 1.35-1.38 |
| **Recently divorced or widowed** | 1.26 | 1.23-1.30 | 1.32 | 1.29-1.36 |
| Recently divorced | 1.27 | 1.23-1.31 | 1.32 | 1.28-1.36 |
| Recently widowed | 1.17 | 0.99-1.38 | 1.35 | 1.15-1.58 |
| **Long-term divorced or widowed** | 1.28 | 1.26-1.29 | 1.37 | 1.36-1.39 |
| Long-term divorced | 1.28 | 1.26-1.29 | 1.38 | 1.36-1.39 |
| Long-term widowed | 1.24 | 1.14-1.34 | 1.29 | 1.20-1.40 |
|  |  |  |  |  |
| *Age 45-64* |  |  |  |  |
| **Entire sample** | 1.20 | 1.20-1.21 | 1.20 | 1.20-1.21 |
| **Recently divorced or widowed** | 1.15 | 1.12-1.18 | 1.17 | 1.14-1.20 |
| Recently divorced | 1.13 | 1.09-1.16 | 1.14 | 1.11-1.18 |
| Recently widowed | 1.21 | 1.14-1.28 | 1.26 | 1.19-1.32 |
| **Long-term divorced or widowed** | 1.21 | 1.20-1.22 | 1.21 | 1.20-1.21 |
| Long-term divorced | 1.21 | 1.21-1.22 | 1.21 | 1.20-1.22 |
| Long-term widowed | 1.14 | 1.12-1.17 | 1.14 | 1.12-1.17 |
|  |  |  |  |  |
| *Age 65-79* |  |  |  |  |
| **Entire sample** | 1.12 | 1.11-1.14 | 1.14 | 1.13-1.16 |
| **Recently divorced or widowed** | 1.19 | 1.12-1.26 | 1.21 | 1.14-1.28 |
| Recently divorced | 1.20 | 1.01-1.42 | 1.25 | 1.06-1.48 |
| Recently widowed | 1.18 | 1.11-1.27 | ^a^ | ^a^ |
| **Long-term divorced or widowed** | 1.12 | 1.10-1.13 | 1.14 | 1.12-1.15 |
| Long-term divorced | 1.12 | 1.10-1.14 | 1.14 | 1.12-1.16 |
| Long-term widowed | 1.12 | 1.10-1.15 | ^a^ | ^a^ |
